# Foreign body in the appendix presenting as acute appendicitis: a case report

**DOI:** 10.1186/s13256-016-0922-7

**Published:** 2016-05-26

**Authors:** Carlson B. Sama, Leopold N. Aminde, Tsi N. Njim, Fru F. Angwafo

**Affiliations:** 1 Islamic Medicalized Health Centre-Babessi and Department of Surgery, Faculty of Health Sciences, University of Buea, Buea, Cameroon; 2 Galactic Corps Research Group (GCRG), Buea, Cameroon; 3 Clinical Research Education, Networking & Consultancy, Douala, Cameroon and, The University of Queensland, School of Public Health, Brisbane, QLD 4006 Australia; 4 Bamenda Regional Hospital and Health and Human Development (2HD) Research Group, Douala, Cameroon; 5 Department of Surgery, University Teaching Hospital Yaoundé and Gynaeco-Obstetric and Paediatric Hospital, Yaoundé, Cameroon

**Keywords:** Foreign body, Condom, Oral sex, Appendicitis

## Abstract

**Background:**

Foreign bodies are a rare cause of appendicitis. In most instances, ingested foreign bodies pass through the alimentary tract asymptomatically. However, those that enter the lumen of the vermiform appendix may not be able to re-enter the colon and may initiate an inflammatory process. We report a case of acute appendicitis induced by an unusual foreign body.

**Case presentation:**

A 26-year-old Sub-Saharan woman presented with right iliac fossa pain and tenderness. She underwent an open appendectomy which revealed a condom fragment within the appendiceal lumen. A detailed retrospective history confirmed accidental ingestion of the condom 2 weeks prior to onset of symptoms.

**Conclusions:**

Although a rare finding, a variety of foreign bodies can be lodged in the appendix and may instigate an inflammatory process. There is a need to increase awareness of the potential dangers of ingested foreign bodies.

## Background

Worldwide, appendicectomy for acute appendicitis is the most common emergency surgical procedure [1, 2]. The ingestion of inedible and indigestible objects is frequent in children; in adults it is a rare condition that often occurs accidentally, or it can occur in patients with mental disorders or in prisoners [3, 4]. The presence of a foreign body (FB) in the appendix, acting as a cause of an inflammatory process, is a very rare event [5]. Here we present a rare case of an appendicectomy indicated for acute appendicitis due to an accidental ingestion of an unusual FB which is apparently the first case of its kind published in the literature.

## Case presentation

A 26-year-old Sub-Saharan woman presented to our unit with a 1-week history of vague colicky lower abdominal pains occasionally radiating to her right iliac fossa (RIF), which progressively increased in intensity over the last 4 days and localized to her RIF. She also developed nausea and anorexia but reported no episodes of vomiting. She took self-prescribed doses of ibuprofen with no relief, prompting a consult at our health unit.

**Fig. 1:**
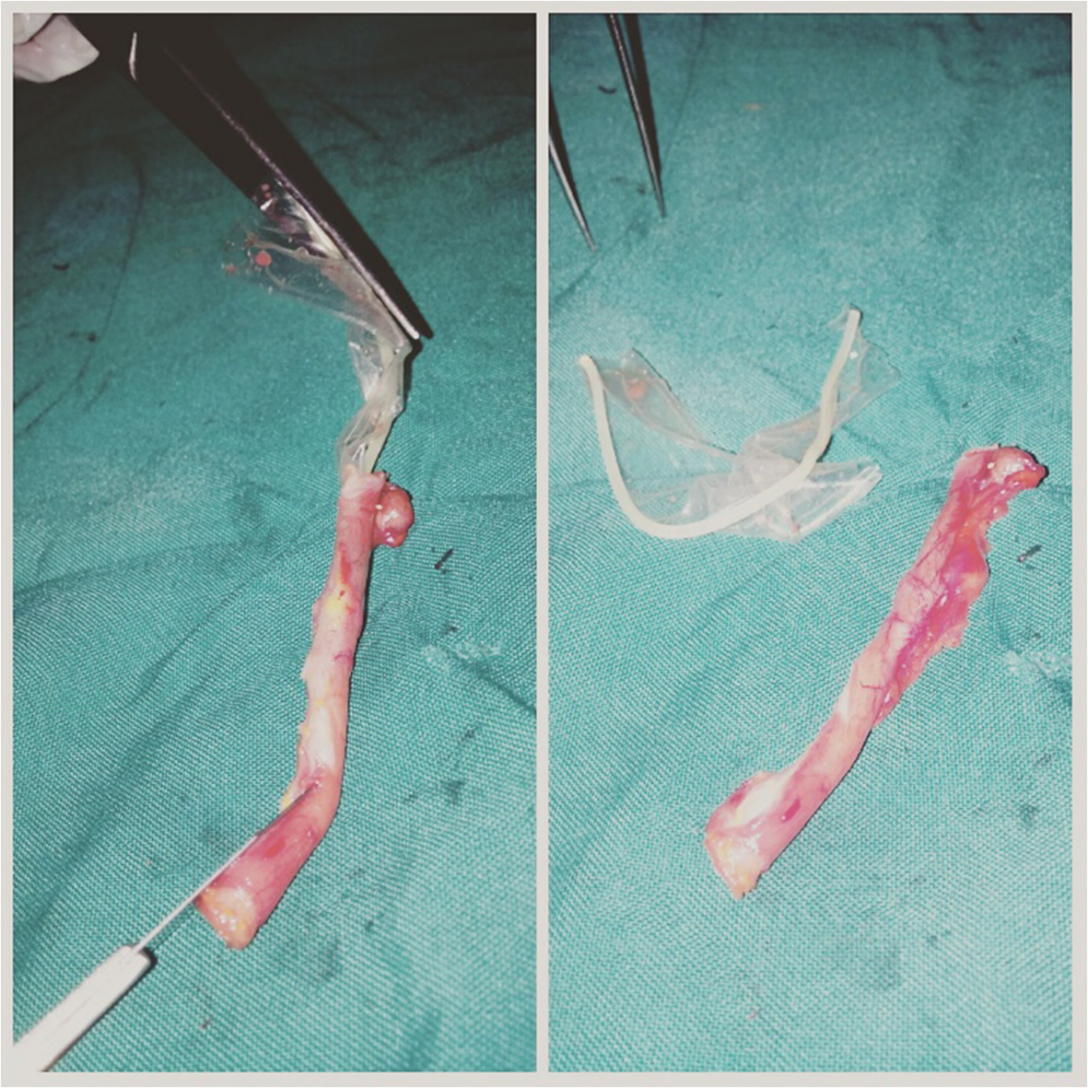
Condom fragment retrieved from appendiceal lumen

On examination, she had a temperature of 38.2 °C, respiratory rate of 14 breaths per minute, pulse of 78 beats per minute, and her blood pressure was 110/82 mmHg. There was tenderness and guarding on palpation of her RIF and a positive Rovsing’s sign. There was no palpable mass and her bowel sounds were normal. A vaginal examination revealed right adnexal tenderness. The rest of the examination was unremarkable. A white blood cell count showed a leucocytosis of 12,500cells/mm^3^ with a neutrophilic predominance of 10,500cells/mm^3^. A working diagnosis of acute appendicitis was suggested based on an Alvarado score of 9/10. An abdominal ultrasound scan was requested which revealed a thickened appendiceal wall and fluid collection around her RIF. No FB was identified. She was prepared for emergency surgery. Via a gridiron incision, her appendix was found to be inflamed and an appendectomy was performed. No intraoperative complication occurred and her abdomen was closed in layers. Due to an unusual feel, we blindly dissected the resected appendix and found an incomplete piece of a rubbery material which was consistent with a condom (Fig. 1). The postoperative period was uneventful and she was discharged on postoperative day 4.

## Discussion

The year 1735 saw the first appendicectomy, which was performed on an 11-year-old boy at St. George’s Hospital in London by Claudius Amyand during which a sewing pin was found to have perforated the appendix [6]. FBs in the appendix are uncommon but well described with an estimated prevalence of 0.0005 % [7, 8]. FBs generally do not cause any complications and pass through the gastrointestinal tract (GIT) spontaneously within a week. The complication rate of ingested FBs is estimated at less than 1 % [8].

Various objects acting as FBs have been reported in the literature to be the cause of acute appendicitis, including bird shot, bullets, fishing lines, screws, coins, stones, toothbrush bristle, pins, needles, teeth, bone fragments, dog hair, fruit seeds and pits, toothpicks, drill bits, tongue studs, crown post, keys and intrauterine contraceptive devices [3, 4, 7–11]. Considering the dependent position of the cecum and to some extent its low motility, such FBs tend to gravitate and settle there. In addition, the chance of entry to the appendiceal lumen is determined not only by its orifice (which might be tight or widely open) but also by the anatomic position of the appendix. There is almost no possibility for a FB to enter the lumen of a retrocecal appendix [8]. Once the FB is lodged in the appendix, peristaltic motion is usually insufficient to expel it back into the cecal lumen. It may remain immobile in the appendix without stimulating an inflammatory process or cause an inflammatory reaction with or without perforation. The period of latency between ingestion of the FB and the onset of symptoms varies from hours to years [4, 9, 11]. Complications usually depend on the size and shape of the FB. Blunt FBs cause appendicitis through obstruction of the appendiceal lumen and remain dormant for longer periods. Elongated sharp FBs are more likely to cause perforations, appendicular abscesses, and peritonitis [4, 8–10].

In our case, there was no history suggestive of FB ingestion during clinical evaluation. However, following retrieval of the condom fragment from the patient’s appendix and its presentation to the patient postoperatively, she could recall having performed oral sex on her boyfriend 2 weeks prior to the onset of symptoms. She reported that the condom had loosened during the act and she accidentally ingested it. She however did not find it necessary to inform us as she did not think it was the cause of her symptoms because she had passed out fragments of the ingested condom in her feces 5 days after ingestion. It is likely that during its migration in the GIT the condom was torn into fragments and a piece was trapped within the vermiform appendix and the rest expelled in fecal matter. Arya *et al*. [12] had reported a case where multiple pieces of a condom were retrieved from the bronchus of a 27-year-old Indian woman. As in the present case report, she inhaled the condom during oral sex.

Apart from accidental ingestion of a condom during oral sex, it is important to note that the act is also associated with several other complications including a significant risk of transmission of human immunodeficiency virus, syphilis, herpes, chlamydia, hepatitis viruses, gonorrhea, and human papilloma virus infections [13].

There is a need to increase awareness of the potential dangers of ingested FBs. Each time such incidents occur, it is advisable patients report promptly for proper evaluation and follow-up until the FB is properly traced as it may account for significant morbidity and mortality.

## Conclusions

Foreign bodies are rare causes of appendicitis. A wide variety of objects can be trapped in the appendix. Our case demonstrates a rare clinical scenario in which an unusual FB became lodged in the appendix and eventually initiated an inflammatory process which resulted in acute appendicitis.
